# Comparative Analysis of Psychological Profiles and Physical Functioning in Addicted and Non-Addicted Male Prisoners: A Pilot Study

**DOI:** 10.3390/jcm14217579

**Published:** 2025-10-25

**Authors:** Michalina Błażkiewicz, Jacek Wąsik, Justyna Kędziorek, Wiktoria Bandura, Jakub Kacprzak, Kamil Radecki, Karolina Radecka, Dariusz Mosler

**Affiliations:** 1 Faculty of Rehabilitation, The Józef Piłsudski University of Physical Education in Warsaw, 00-968 Warsaw, Poland; justyna.kedziorek@awf.edu.pl (J.K.); wiktoria.bandura@awf.edu.pl (W.B.); 2 Institute of Physical Culture Sciences, Jan Długosz University in Częstochowa, 42-200 Częstochowa, Poland; jakub.kacprzak@doktorant.ujd.edu.pl (J.K.); kamil_radecki@onet.pl (K.R.); kowalewskamaja@wp.pl (K.R.); d.mosler@ujd.edu.pl (D.M.)

**Keywords:** substance dependence, prison population, mental health, physical health

## Abstract

**Background/Objectives**: The prison environment presents a unique context for examining the impact of addiction on physical and psychological functioning. Individuals with substance use disorders (SUDs) are overrepresented in correctional facilities and often experience greater emotional difficulties and impaired physical capacity. This study aimed to conduct a comparative analysis of psychological and functional profiles between addicted and non-addicted male inmates in a semi-open correctional facility. **Methods**: The study included 47 male prisoners (19 addicted, 28 non-addicted). Physical performance was assessed using the Countermovement Jump (CMJ), handgrip strength, the Functional Movement Screen (FMS), and the FitLight reaction time test. Psychological functioning was evaluated using six standardized questionnaires: problem-focused, emotion-focused, and avoidant coping strategies, depression (PHQ-9), perceived stress (PSS-10), and self-compassion (SCS). **Results**: No statistically significant differences (*p* > 0.05) were found between addicted and non-addicted inmates in physical performance parameters. Addicted individuals demonstrated slightly higher handgrip strength with lower variability, while non-addicted inmates showed slightly better lower-body power in the CMJ test. Functional movement quality and reaction speed were similar between groups. Psychological assessments also revealed no significant differences between the groups. Coping styles, depressive symptoms, perceived stress levels, and self-criticism scores were comparable in both populations. In the addicted group, deeper squats correlated with lower stress (rho = −0.46, *p* = 0.047), and better hurdle step performance correlated with emotion-focused coping (rho = 0.46, *p* = 0.048). **Conclusions**: Although no statistically significant differences were found between addicted and non-addicted male inmates in the assessed physical and psychological outcomes, the limited sample size and context-specific nature of this pilot study suggest that these findings should be viewed as preliminary and interpreted with caution. Nonetheless, the observed associations between physical performance and psychological variables indicate subtle interconnections between motor capacity, stress perception, and coping mechanisms that merit further investigation in larger, longitudinal studies.

## 1. Introduction

The prison environment is a unique and highly structured setting that presents a wide range of psychological and physiological challenges for incarcerated individuals [1]. The intersection of incarceration and substance use is a particularly critical concern in public health and correctional systems worldwide. Research consistently shows that individuals with substance use disorders (SUDs) are significantly overrepresented in prison populations, often experiencing more complex mental health problems, impaired coping strategies, and reduced physical well-being compared to the general population and their non-addicted peers [2,3,4].

Addiction is not merely a behavioral issue—it is a multifaceted disorder that affects cognitive function, emotional regulation, social behavior, and physical health. Incarcerated individuals with a history of addiction are frequently characterized by higher levels of impulsivity, emotional dysregulation, depression, and stress sensitivity, all of which can negatively impact rehabilitation outcomes and contribute to higher recidivism rates [5,6,7]. In addition to these psychological difficulties, addiction often coincides with deteriorated physical health, motor deficiencies, and poor functional performance due to long-term substance-related physiological damage or lifestyle factors [2].

While the psychological consequences of addiction in prison populations have been extensively documented, the literature addressing the interplay between chemical dependence and objective physical performance remains limited. Most studies either focus solely on mental health outcomes or broadly report on general physical health without precise measurement of functional capacities such as muscle strength, movement efficiency, or reaction speed. This represents a critical gap because impaired physical performance may exacerbate psychological vulnerabilities, reduce participation in rehabilitation programs, and limit overall reintegration potential.

Despite the wealth of literature on the psychological and physical consequences of addiction, relatively few studies have examined these domains simultaneously within prison populations. Even within studies addressing physical functioning, there is little systematic comparison between addicted and non-addicted inmates within the same institutional context. This gap is particularly relevant because differences in physical performance may not merely reflect historical substance use but also interact with coping strategies, stress resilience, and the capacity to engage in structured rehabilitation programs.

A multidimensional approach—one that considers both mental and physical domains—is crucial to developing individualized rehabilitation programs that are tailored to the specific needs of inmates. By explicitly linking addiction history to both psychological and functional performance, research can move beyond descriptive accounts to generate actionable insights into how deficits in one domain may influence outcomes in the other. Understanding these relationships may enhance intervention strategies, promote positive lifestyle change, and improve long-term quality of life [8].

Importantly, in this study, “physical functioning” refers specifically to measurable aspects of physical performance, such as muscle strength, movement efficiency, and reaction time, rather than general physical health. This study addresses this gap by conducting a comprehensive comparison of psychological profiles and physical functioning between addicted and non-addicted male prisoners in a semi-open correctional facility. The psychological component includes measures of coping strategies (problem-focused, emotion-focused, and avoidant), perceived stress, depressive symptoms, and self-compassion—factors known to influence emotional resilience, behavior regulation, and recovery potential [9,10,11]. These tools allow for an in-depth evaluation of how inmates manage stress and internal distress and whether differences emerge based on addiction history.

Simultaneously, physical functioning was assessed using a battery of standardized tests, including the Functional Movement Screen (FMS) for motor control and asymmetries, handgrip strength as a marker of general muscular strength and health status, Countermovement Jump (CMJ) for lower-body power, gait analysis for dynamic movement efficiency, and FitLight reaction testing to measure cognitive-motor coordination and response speed [12,13]. These assessments are supported by previous research linking physical performance to overall well-being [14], mental health, and functional capacity, particularly in correctional and clinical populations [15,16], and in youth or longitudinal populations [17].

The combination of these tools offers a holistic view of the participants’ functioning. Moreover, the use of objective, technology-supported assessments, such as the FitLight Trainer system and BTS G-Sensor IMU, ensures the precision and replicability of the measurements.

The primary aim of this study was to investigate whether there are significant differences in psychological and physical profiles between addicted and non-addicted inmates, and to explore potential associations between these domains. By explicitly addressing the underexplored link between chemical dependence and functional performance, this study moves beyond descriptive comparisons to identify specific deficits that may inform targeted rehabilitation strategies. A secondary aim was to generate practical insights for correctional health professionals, therapists, and rehabilitation program developers. By identifying specific deficits or strengths in each group, this research may guide the development of targeted interventions that support both mental and physical rehabilitation within the correctional system, potentially reducing relapse and reoffending upon release.

## 2. Materials and Methods

### 2.1. Study Participant Overview

The research was conducted on two separate dates (21 and 28 March 2025) within a semi-open correctional facility. A total of 47 incarcerated males participated, divided into two groups: individuals with a history of substance dependence (*n* = 19) and those without such a history (*n* = 28). The addicted group included inmates at various stages of substance treatment: awaiting therapy, actively participating in it, or having completed it. Their addictions involved alcohol, psychoactive substances, or both (Table 1). In this manuscript, the term “addiction” is used to describe participants with a history of compulsive substance use. While it is acknowledged that some participants may have met formal Diagnostic and Statistical Manual of Mental Disorders (DSM) [18] criteria for substance use disorder, complete diagnostic records were not always available. For clarity and consistency, the term “addiction” has been retained throughout the text.

No statistically significant differences were found between the groups regarding age, body mass, body height, total time spent in prison, or time spent in the semi-open prison.

The study enrolled male inmates residing in a semi-open prison, all over 18 years of age, physically capable of performing tasks in a standing position, and free from diagnosed neurological conditions. Only individuals who had maintained abstinence for a minimum of 3 months, verified through prison records and self-report, and who were in a stable stage of therapy or rehabilitation (defined as ongoing abstinence, consistent engagement in therapeutic activities, and clinical assessment indicating absence of acute psychological or behavioral instability) were eligible for inclusion. Potential confounders, including age, type and duration of addiction, length of incarceration, and level of habitual physical activity, were not controlled for in inferential analyses. Exclusion criteria comprised refusal to participate or the presence of any contraindications for standing balance assessment. Additionally, individuals with known neurological disorders, such as Parkinson’s disease or epilepsy, as well as those experiencing acute psychiatric episodes during the testing period, were not included. At the time of data collection, none of the participants were involved in structured balance or motor rehabilitation programs. Before incarceration, all participants reported engaging in recreational (amateur) physical activity, typically gym-based training approximately twice per week, and none had participated in sport at an advanced or professional level. At the time of assessment, participants had no diagnosed psychiatric disorders that would contraindicate participation; all individuals were screened and approved by prison medical and psychological staff and therefore met the inclusion criteria.

#### 2.1.1. Participant Demographics and Group-Specific Characteristics

**General Characteristics**

Table 1 summarizes the demographic, anthropometric, and incarceration characteristics of the study participants, divided into the addicted and non-addicted groups. No statistically significant differences were observed between groups regarding age, body mass, height, or duration of imprisonment.

**Addiction Characteristics**

Among the 19 participants with a history of substance dependence, most were alcohol-dependent (*n* = 12), six used psychoactive substances, and one had both alcohol and drug addiction. The duration of substance use ranged from 2 to nearly 30 years. At the time of the study, one participant was undergoing therapy, 13 had completed treatment, one was awaiting therapy, and three were abstinent without current intervention.

Alcohol-dependent inmates most often reported long-term use (3–30 years). The OT Wojkowice treatment center was the primary treatment center where most participants completed therapy and showed moderate to high engagement. Some attended therapy at the OT Jasło center or in outpatient programs. Others maintained abstinence or engaged in peer support initiatives such as Alcoholics Anonymous and the “*Alcohol—Enemy of Roads*” program.

Among those addicted to psychoactive substances (α-PVP, amphetamines, cannabinoids), the average duration of use was 2–5 years. Most had completed therapy or were abstinent, while one awaited court-ordered treatment. The participant with combined alcohol and drug dependence had completed therapy at OT Wojkowice and continued attending Alcoholics Anonymous meetings (Table 2).

#### 2.1.2. Power Analysis

A post hoc statistical power analysis was performed using G*Power (version 3.1.9.7; Kiel University, Kiel, Germany) [19] to assess the adequacy of the sample size (*n* = 47). A large effect size (Cohen’s d = 0.8) was defined according to Cohen [20]. To provide a clearer overview of the study’s sensitivity, achieved power (1 − β) was also estimated for medium (d = 0.5) and small (d = 0.3) effects based on the sample allocation (n1 = 28, n2 = 19). The corresponding power values were 0.75 for a large effect, 0.38 for a medium effect, and 0.17 for a small effect. These results indicate that adequate sensitivity was achieved only for large effects, whereas the study was underpowered to detect medium or small effects.

#### 2.1.3. Ethical Considerations

Ethical approval for this study was granted by the University Institutional Review Board (Reference No. SKE01-15/2023), and the research was conducted following the Declaration of Helsinki’s ethical guidelines. Before participation, all inmates received comprehensive verbal and written information about the study’s purpose, procedures, and potential risks and benefits. They were informed of their right to refuse or withdraw from the study at any stage without any penalty or impact on their legal or institutional status. Written informed consent was obtained from each participant before data collection. Confidentiality and anonymity were strictly maintained, with all data coded to prevent identification of individual participants. Participation in the study was entirely voluntary.

### 2.2. Measurement Protocols

Participants underwent a series of five distinct tests designed to assess various aspects of reaction time, psychological states, and physical function. The physical assessments included the Functional Movement Screen (FMS), Handgrip Strength, and Countermovement Jump (CMJ). All procedures were conducted following standardized protocols, and cultural validity of the scales for the Polish population was considered where applicable. Participant comfort and safety were prioritized throughout.

A BTS G-Sensor (BTS Bioengineering S.p.A., Garbagnate Milanese, Italy), a wireless inertial measurement unit (IMU), served as the instrument for collecting kinematic data during the Countermovement Jump (CMJ). This device is optimized for kinematic assessment in both clinical and research contexts. Its compact form factor (37 g, 70 mm × 40 mm × 18 mm) integrates high-resolution triaxial inertial sensors, including an accelerometer (16-bit per axis, settable sensitivity up to ±16 g), a gyroscope (16-bit per axis, adjustable range up to ±2000 deg/s), and a magnetometer (13-bit, ±1200 µT range). For non-invasive trunk kinematic measurement, the sensor was firmly attached to the participant’s waist using a semi-elastic belt. Data were captured at a frequency of 100 Hz and wirelessly sent via Bluetooth^®^ to a dedicated computer for real-time visualization and subsequent processing.

#### 2.2.1. Countermovement Jump (CMJ)

The Countermovement Jump (CMJ) was used to assess lower body power. Participants stood with hands placed on their hips to isolate lower body movement. They were instructed to perform a rapid downward movement immediately followed by a maximal vertical jump. Participants were instructed to land softly. Due to logistical restrictions inherent to the prison environment, such as limited testing time, strict supervision requirements, and the need to minimize testing duration for safety and compliance reasons, only one CMJ trial was performed per participant. Nevertheless, previous research has demonstrated that single-trial CMJ assessments can yield reliable and valid results when standardized procedures and validated equipment are used [21,22]. Key parameters such as jump height (calculated from flight time), peak power, and velocity peak were taken for analysis. The CMJ protocol follows previously validated procedures for reliability and reproducibility in research contexts [21].

#### 2.2.2. Functional Movement Screen (FMS)

The Functional Movement Screen (FMS) was conducted to evaluate fundamental movement patterns and identify any limitations or asymmetries. The FMS comprises seven standardized movements: deep squat, hurdle step, in-line lunge, shoulder mobility, active straight-leg raise, trunk stability push-up, and rotational stability.

Specifically, the deep squat assesses overall mobility and stability of the hips, knees, and ankles during a full squat. The hurdle step evaluates dynamic balance and single-leg stability as the participant steps over an obstacle. The in-line lunge tests hip and ankle mobility along with trunk stability during a forward lunge performed in a straight line. Shoulder mobility measures the ability to reach behind the back, reflecting shoulder flexibility and range of motion. The active straight-leg raise assesses hamstring flexibility and core stability by raising a straight leg while lying down. The composite raw score summarizes overall movement quality and functional mobility based on all FMS tests.

Each movement was scored on an ordinal scale from 0 to 3, where 0 indicated pain during the movement, 1 represented inability to perform the movement, 2 indicated performance with compensation, and 3 denoted perfect execution. A composite score ranging from 0 to 21 was calculated by summing the individual scores. The assessment was carried out using the original FMS test kit to ensure standardization and accuracy. The FMS has been previously validated and widely used internationally [23,24]. Standardized procedures and clear demonstrations ensured reliability. Before testing, participants received clear instructions and demonstrations, and two trials were allowed for each movement, with the highest score recorded.

#### 2.2.3. Handgrip Strength

Handgrip strength was measured using a hydraulic hand dynamometer (Saehan Corporation, Model SH5001, Changwon, Republic of Korea). Two distinct positions were tested: one with the arm extended straight alongside the torso, and another with the elbow flexed at a 90-degree angle. For each position, participants were instructed to squeeze the dynamometer with maximal effort for three seconds. Two trials were performed in each position on each hand (dominant and non-dominant) with a rest period of at least 30 s between trials. The highest reading obtained from any of the trials for each hand, measured in kilograms (kg), was recorded for analysis. The measurement follows standardized dynamometry procedures validated in adult populations, including European cohorts [25].

#### 2.2.4. Fitlight Reaction Speed

Reaction time in this study was assessed using the FitLight Trainer system (FitLight Sports Corp., Kingston, ON, Canada), which features eight wireless, interactive lights that can be positioned in various arrangements and activate in randomized sequences to evaluate response speed. Participants were instructed to react as quickly as possible by deactivating the illuminated lights, through touch or pressing. For this protocol, participants stood in front of wall-mounted lights, with all LEDs positioned within easy reach of their hands, eliminating the need to lean forward. During the 60 s task, participants responded to the light cues by touching the active lights. The dedicated software recorded both the reaction time—defined as the interval between light activation and the participant’s response—and the total number of lights successfully deactivated. The protocol was designed to challenge quick decision-making and motor response. Moreover, this method has been validated for measuring sensorimotor response and cognitive-motor integration [26].

#### 2.2.5. Psychological Assessment

To evaluate the psychological characteristics of participants, six standardized tests were administered: Problem-Focused Coping, Emotion-Focused Coping, Avoidant Coping, Self-Compassion Scale (SCS), Patient Health Questionnaire-9 (PHQ-9), and Perceived Stress Scale (PSS-10).

- Problem-Focused Coping, Emotion-Focused Coping, and Avoidant Coping are subscales derived from widely used coping inventories, such as the COPE Inventory. These scales assess the strategies individuals use to manage stress. Problem-focused coping involves actively dealing with the source of stress, emotion-focused coping reflects strategies to manage emotional distress, and avoidance coping involves denial, distraction, or shutting oneself off from the problem [27]. Polish-language versions of these scales have been validated for use in the Polish population, including institutionalized and correctional settings [28].
- The Self-Compassion Scale (SCS) measures how individuals relate to themselves during difficult times, which includes aspects such as self-compassion, simple humanity, and mindfulness. Higher scores indicate greater self-compassion, which is linked to better emotional regulation and resilience [9]. The SCS has been culturally adapted and validated for Polish respondents [29].
- The Patient Health Questionnaire-9 (PHQ-9) is a brief diagnostic tool used to screen for symptoms of depression based on DSM criteria. It consists of nine items and provides a severity score to evaluate depressive symptomatology [30]. Polish-language adaptations of the PHQ-9 have been validated in both clinical and non-clinical populations [31].
- The Perceived Stress Scale (PSS-10) assesses the degree to which individuals perceive situations in their lives as stressful. It captures feelings of unpredictability, uncontrollability, and overload in the past month [11]. The PSS-10 has been validated for use in Polish populations, including correctional samples [32].

All psychological assessments were administered following their standardized protocols, with clear instructions and demonstrations provided to ensure consistency. Where available, culturally and linguistically validated Polish versions of the instruments were used. Participants completed the self-report questionnaires in a quiet environment, with sufficient time allowed and assistance offered for any questions. This standardized approach ensures reliable and culturally appropriate measurement of psychological constructs in the Polish prison population. The collected data were used to construct a multidimensional psychological profile for both the addicted and non-addicted groups.

### 2.3. Statistical Analysis

Statistical analysis was conducted using Statistica v.12 (StatSoft, Tulsa, OK, USA), with the significance level set to *p* < 0.05. The normality of distribution for all parameters was assessed using the Shapiro–Wilk test. The Mann–Whitney U test was employed to determine whether significant differences existed between the addicted and non-addicted groups. To compute the effect size for a U Mann–Whitney test, the following formula was used: $r=\frac{Z}{\sqrt{N}}$, where Z is the Z-score from the test, and N is the total number of observations. In this case, N = 47. The effect size interpretation was as follows: r < 0.3—small effect, 0.3 ≤ r ≤ 0.5—medium effect, and r > 0.5—large effect [33]. In this study, multiple Mann–Whitney U tests were performed across several physical and psychological outcomes. No correction for multiple comparisons was applied, as the small sample size and exploratory design of this pilot study could render such corrections overly conservative and increase the risk of type II errors, potentially obscuring meaningful trends. Therefore, uncorrected *p*-values are reported to allow the detection of preliminary associations warranting further investigation.

In addition to group comparisons, Spearman’s rank-order correlation tests were conducted to examine associations between physical performance measures (CMJ, FMS, handgrip strength, FitLight reaction time) and psychological measures (coping strategies, SCS, PHQ-9, PSS-10) within the addicted and non-addicted groups. Correlation coefficients (rho) and significance values (*p*) were calculated to assess the strength and direction of relationships. The correlation strength was interpreted as follows: 0–0.19 indicates a very weak or negligible relationship, 0.20–0.39 a weak relationship, 0.40–0.59 a moderate relationship, 0.60–0.79 a strong relationship, and values above 0.80 a very strong relationship [34].

For the principal Spearman correlations, 95% confidence intervals (CIs) were computed using the Fisher z-transformation to improve the accuracy and interpretability of correlation estimates [35,36]. The Fisher z-transformation was calculated as

$$z^{\prime }=0.5\times ln\left(\frac{1+rho}{1-rho}\right).$$

The standard error (*SE*) of the transformed value was estimated as $SE=1/\sqrt{n-3}$, where *n* is the sample size. The 95% confidence limits for the transformed correlation were then obtained as: ${z\prime }_{lower,\;upper}=z^{\prime }\pm 1.96\times SE,$ and subsequently back-transformed to the correlation scale using: ${rho}_{lower,upper}=\frac{e^{2z^{\prime }}-1}{e^{2z^{\prime }}+1}$. This approach allows for precise estimation of the uncertainty surrounding the correlation coefficient, even with small samples typical of exploratory or pilot research. Wider confidence intervals reflect greater uncertainty and should therefore be interpreted cautiously, particularly in the presence of non-significant results.

## 3. Results

### 3.1. Countermovement Jump (CMJ)

Jump performance parameters are summarized in Table 3, showing small and non-significant differences between non-addicted and addicted individuals (all *p* > 0.05).

The non-addicted group exhibited a slightly higher median jump height (25.9 cm vs. 22.7 cm) and a wider interquartile range, suggesting marginally greater variability and potential for peak performance. Peak power and velocity were comparable between groups, with only minor differences in the upper and lower quartiles. Overall, effect sizes were small (0.08–0.14), indicating minimal practical differences in explosive lower-body performance between groups.

### 3.2. Functional Movement Screen (FMS)

FMS performance parameters are presented in Table 4. Across most individual tests, non-addicted and addicted participants showed similar median scores, indicating comparable fundamental movement patterns, flexibility, and stability.

Slight trends favoring non-addicted individuals were observed in the In-Line Lunge (left leg) and shoulder mobility; however, only the left-hand reach and the composite FMS raw score reached statistical significance, with moderate effect sizes (Z = 2.36, r = 0.34 and Z = 2.25, r = 0.33, respectively). These findings suggest modestly better shoulder flexibility and overall movement quality in the non-addicted group, while most other FMS parameters showed minimal or non-significant differences.

### 3.3. Handgrip Strength

Across all positions, the addicted group demonstrated slightly higher and more consistent handgrip strength values compared to non-addicted participants; however, none of these differences reached statistical significance (all *p* > 0.05). This trend may suggest marginally better neuromuscular efficiency or physical conditioning among addicted individuals, though further research is required to confirm this observation (Table 5).

### 3.4. Fitlight Reaction Speed

The Fitlight Reaction Speed results show minor, non-significant differences between non-addicted and addicted individuals in reaction time and the number of switched-off lights (Table 6). Addicted individuals generally have slightly slower reaction times, with a higher Q1 value and a marginally higher median compared to non-addicted participants. However, the difference is minimal, and the upper quartile values are nearly the same. In terms of the number of switched-off lights, non-addicted individuals show slightly better performance at the median and upper quartile levels, indicating they were able to respond to more stimuli within the test period. Overall, the results suggest that while reaction time is only slightly slower in addicted individuals, their ability to respond effectively to multiple stimuli may be somewhat reduced.

### 3.5. Psychological Assessment

Across all six psychological measures (Table 7), no statistically significant differences were observed between non-addicted and addicted participants. Median scores and interquartile ranges were closely aligned for problem-focused coping, emotion-focused coping, avoidant coping, self-critical attitudes (SCS), depressive symptoms (PHQ-9), and perceived stress (PSS-10). Although avoidant coping showed a slight trend toward higher scores in the addicted group, this difference was not significant (*p* = 0.05, r = −0.28). Overall, these findings suggest that addiction status alone does not substantially affect coping strategies, self-criticism, depressive symptoms, or perceived stress within this incarcerated sample.

### 3.6. Associations Between Physical Performance and Psychological Variables in Addicted and Non-Addicted Groups

Spearman’s rank-order correlations were calculated to explore the relationships between physical performance measures (functional movement, strength, jump performance, and reaction time) and psychological variables (coping strategies, self-control, depressive symptoms, and perceived stress) in the addicted (*n* = 19) and non-addicted groups (*n* = 28) (Table 8).

In the addicted group, better performance in certain functional movement and strength tests was linked with more favorable psychological outcomes. Specifically, deeper squats were associated with lower perceived stress, and improved hurdle step performance on the left leg was related to greater use of emotion-focused coping. Interestingly, right-hand grip strength showed opposite associations with depressive symptoms across groups: higher strength was related to more depressive symptoms in the addicted group, whereas in the non-addicted group, greater strength was associated with fewer depressive symptoms. Additionally, trends suggested that stronger or faster jump performance was linked to lower avoidant coping, and slower reaction time was associated with greater avoidant coping tendencies, though these did not reach conventional significance levels.

Overall, the inclusion of 95% confidence intervals provides additional insight into the robustness of these associations. Most intervals were relatively wide, reflecting the small sample size and exploratory nature of the study; thus, observed correlations should be interpreted as preliminary patterns that warrant confirmation in larger, more powered samples.

## 4. Discussion

The primary aim of this study was to assess whether differences exist in physical and psychological functioning between addicted and non-addicted inmates and to examine associations between these domains. Across all measures, including Countermovement Jump (CMJ), Functional Movement Screen (FMS), Handgrip Strength, reaction speed, and psychological functioning, no statistically significant differences were observed. However, correlation analyses revealed associations between physical performance and psychological variables, particularly in the addicted group, highlighting nuanced links between motor function and coping or stress outcomes. This lack of statistically significant differentiation, considering the pilot and context-specific nature of this study, suggests preliminary trends rather than definitive conclusions, raising important questions about the impact of addiction and incarceration on physical and psychological outcomes in this population.

### 4.1. Physical Assessments

For CMJ, FMS, and handgrip strength, performance was broadly similar between groups. Slight variations in jump height or FMS lunge scores did not reach clinical significance. Handgrip strength in the addicted group was marginally higher, but this finding is speculative and may reflect individual variability rather than a systematic effect of addiction. International literature suggests that basic neuromuscular function and gross motor performance may be relatively preserved in adult male prisoners, particularly when inmates engage in regular recreational or therapeutic physical activity [22,37,38,39,40,41]. This highlights the potential role of the structured prison environment, which may provide opportunities for physical activity that mitigate the negative physical effects of chronic substance use.

Furthermore, Spearman’s correlations indicated that deeper squats were associated with lower perceived stress in the addicted group (rho = −0.46, *p* = 0.047), and improved left-leg hurdle step performance correlated with greater use of emotion-focused coping (rho = 0.46, *p* = 0.048). Trends also suggested that stronger/faster jump performance was linked to lower avoidant coping (rho = −0.41, *p* = 0.078), and slower reaction time was associated with more avoidant coping (rho = 0.45, *p* = 0.051).

Reaction time measured via the FitLight system showed negligible differences between groups, with both performing within normative ranges [26,42,43,44,45]. While chronic substance use is often associated with deficits in executive function and attention, the simplicity of this sensorimotor task may have limited the ability to detect subtle cognitive impairments, particularly in a prison context where basic motor responses remain intact.

### 4.2. Psychological Assessment

No significant differences were found in coping strategies, self-criticism, depressive symptoms, or perceived stress. Several factors may explain this null effect. First, the self-reported nature of the assessments introduces potential biases, as prisoners may underreport stress or depressive symptoms due to stigma or concerns about institutional repercussions [27]. Second, the standardized tests applied, while validated for the Polish population, may not fully capture context-specific stressors or emotional dysregulation in prison environments [46,47]. Third, participants in both groups had maintained abstinence and were at various stages of rehabilitation, which may have attenuated psychological differences.

Interestingly, right-hand grip strength showed opposite associations with depressive symptoms across groups: higher strength was associated with more depressive symptoms in the addicted group (rho = 0.47, *p* = 0.043, 95% CI [0.02, 0.76]) and fewer depressive symptoms in the non-addicted group (rho = −0.46, *p* = 0.049, 95% CI [−0.71, −0.10]). This suggests that physical capacity may interact differently with mental health indicators depending on addiction history. The relatively wide confidence intervals indicate considerable uncertainty around these effect estimates, reflecting the limited sample size, particularly within the addicted subgroup (*n* = 19). Therefore, these relationships should be interpreted as preliminary trends requiring confirmation in larger, adequately powered studies.

These findings contrast with some international studies reporting higher emotional dysregulation and maladaptive coping among substance-dependent populations [20,48]. However, they align with research suggesting that structured environments, social support, and rehabilitation programs can buffer against psychological impairments, even in individuals with a history of substance use. Thus, the prison context and rehabilitation processes themselves may contribute to a leveling effect, mitigating expected differences between addicted and non-addicted inmates.

### 4.3. Explanations for Null Findings and Implications

The absence of significant differences between addicted and non-addicted inmates may be explained by several factors. First, the relatively small sample size (*n* = 47) may have limited the statistical power needed to detect subtle effects, suggesting that future studies should include larger cohorts to enhance sensitivity and generalizability. Second, unmeasured confounding variables, such as differences in age, type and duration of addiction, length of incarceration, prior physical activity, treatment phase, nutrition, and comorbid medical or psychological conditions, may have obscured potential group differences. Given the small sample size, formal multivariate regression analyses were not feasible; however, descriptive stratified analyses by substance type and therapeutic stage were performed and are reported above. These analyses are exploratory and highlight potential trends that warrant confirmation in larger studies. Finally, a true null effect cannot be ruled out; it is possible that within the structured environment of a semi-open prison, where inmates have access to rehabilitation programs and regular recreational activity, a history of addiction does not substantially impair overall physical performance or basic psychological functioning.

Nonetheless, the observed correlations indicate that individual differences in physical performance may still reflect or influence psychological outcomes, particularly in the addicted group.

### 4.4. Practical and Theoretical Implications

These results suggest that prison-based rehabilitation and physical activity programs may attenuate the expected negative effects of substance dependence. From a practical standpoint, correctional healthcare providers and rehabilitation staff should continue to promote structured physical activity and psychological support, as these interventions may help preserve function across both addicted and non-addicted populations. Theoretically, the findings challenge assumptions that addiction inevitably leads to broad deficits in physical and basic psychological functioning, highlighting the importance of context and the recovery phase in shaping outcomes.

Moreover, the links between functional movement, strength, and coping strategies underscore that physical training could play a supportive role in psychological rehabilitation, particularly for inmates with a history of substance use.

Future research should employ larger samples, longitudinal designs, more sensitive cognitive and motor assessments, and control for confounders such as physical activity levels, nutrition, and substance type.

## 5. Study Limitation

Several limitations should be noted. First, this investigation was conducted as a pilot study with a relatively small sample size (*n* = 47), which limits statistical power and increases the risk of type II error; therefore, null findings should be interpreted with caution. Second, the cross-sectional design restricts causal inferences and temporal assessments of how addiction or incarceration duration impacts physical and psychological outcomes. Third, the heterogeneous nature of the addicted group, including individuals at different stages of addiction treatment, may have introduced variability in the results. Future studies should stratify participants based on treatment status or addiction severity to clarify these effects. Fourth, the reliance on self-reported psychological measures may be subject to social desirability or reporting biases, particularly in a prison environment where stigma and mistrust are prevalent [49]. Fifth, only male inmates were included, limiting applicability to female incarcerated populations who may exhibit different patterns of addiction and health outcomes [50]. Finally, other potentially confounding variables, including age, type and duration of addiction, length of incarceration, level of habitual physical activity, nutrition, comorbid medical conditions, and treatment stage, were not controlled for and could influence the results. The small sample size precluded multivariate regression analyses; therefore, the reported stratified analyses by substance type and therapeutic stage are exploratory and should be interpreted with caution.

In addition, although descriptive stratified analyses were performed, the small sample size (*n* = 47) precluded formal multivariate regression modeling because of the risk of overfitting. The post hoc power analysis showed that the study was only sufficiently powered to detect large effects (d ≈ 0.8) and underpowered for medium-to-small effects; therefore, nonsignificant results should be interpreted with caution. Confidence intervals for correlations indicate wide uncertainty for several estimates, particularly in the smaller addicted subgroup (*n* = 19).

Overall, because this is a pilot, context-bound study with limited power, the findings should be regarded as exploratory and hypothesis-generating rather than conclusive or directly generalizable to other prison populations or settings. Future studies should stratify participants based on treatment status, duration of abstinence, and addiction severity to clarify these effects.

## 6. Conclusions

In this pilot study of a single semi-open correctional facility, no statistically significant differences were observed between addicted and non-addicted male inmates in the assessed physical performance and psychological measures. However, due to the limited sample size, cross-sectional design, and absence of adjusted analyses controlling for potential confounders (such as age, type and duration of addiction, length of incarceration, and level of habitual physical activity), the robustness of these conclusions is limited.

Descriptive stratified analyses by substance type (alcohol vs. drugs) and therapeutic stage (awaiting therapy, ongoing, completed, abstinent) were performed to explore potential trends. While some minor variations were observed, these findings are exploratory and should be interpreted cautiously.

Observed correlations between aspects of physical performance (e.g., functional movement, handgrip strength, and CMJ) and psychological variables (coping strategies, stress perception, and depressive symptoms) highlight potentially meaningful relationships that may differ depending on addiction history. These findings underscore the importance of considering both physical and psychological domains in rehabilitation planning.

Overall, these results should be regarded as preliminary trends rather than definitive evidence that addiction history does not influence physical or psychological outcomes. Confirmation through larger, multi-site, and longitudinal studies, with appropriate control for confounders and potentially multivariate analyses, is necessary to verify or refine the patterns identified in this study.

## Figures and Tables

**Table 1:** Demographic, Anthropometric, And Incarceration Characteristics of Study Participants by Group.

Variable	All Participants (*n* = 47)	Non-Addicted (*n* = 28)	Addicted (*n* = 19)
Age [years], Median (IQR)	24.3 (20.1–29.4)	26.5 (21.1–30)	21.6 (19.6–24.3)
Body Mass [kg], Median (IQR)	82.9 (74.5–93.3)	81.5 (74.3–91)	86.7 (78.7–94.4)
Height [cm], Median (IQR)	178 (175–184)	178.5 (176–185)	177 (173–183)
Time in Prison [months], Median (IQR)	9 (3.5–18)	8 (3.25–17.25)	10 (5.5–19)
Time in Semi-Open Unit [months], Median (IQR)	4 (1.5–9)	3.5 (1.5–6.5)	7 (1–10)
Secondary Education [%]	34	39	26
Vocational Education [%]	34	32	36
Primary Education [%]	27	25	31
Higher Education [%]	4	3	5
Single [%]	57	67	42
Married [%]	14	21	5
Divorced [%]	21	7	42
Domestic Partnership [%]	6	3	10

**Table 2:** Addiction Characteristics and Treatment Outcomes among Incarcerated Individuals.

Type of Addiction	Estimated Duration of Use	Therapy Status	Facility/Location	Engagement Level	Additional Notes
Alcohol	~11 years	Ongoing	OT Wojkowice	Not documented	Currently receiving therapy
	~14 years	Completed	OT Wojkowice	High	
	~16 years	Completed	OT Wojkowice	High	
	Several years	Completed	OT Wojkowice	High	
	~10 years	Completed	OT Wojkowice	High	
	~20 years (2005–2025)	Completed	OT Wojkowice	Moderate	
	~4 years	Completed	OT Jasło	Moderate	
	~10 years + relapse	Completed	Not specified	Moderate	Previously abstinent for 10 years
	~29 years	Abstinent	Not applicable	Not documented	Six years of confirmed abstinence; no prison therapy
	~11 years	Completed	Not specified	Low	Limited behavioral change observed
	~3 years	Outpatient (2020)	Not applicable	Not documented	Participated in post-treatment reintegration program
	~10 years	Completed	OT Wojkowice	Not documented	
Drugs	~5 years	Completed	Not specified	Moderate	
	~2 years	Awaiting therapy	OT Wojkowice		Court-mandated; scheduled for November 2025
	~3 years (estimated)	Abstinent	Not applicable		Ended drug use prior to incarceration
α-PVP	~2 years	Completed	OT Suwałki	High	
Amphetamine	~5 years	Completed	OT Przemyśl	High	Treatment followed relapse
Cannabinoids	~5 years	Abstinent	Not applicable		Drug-free for 1.5 years; completed a prevention program
Alcohol + Drug	Various	Completed	OT Wojkowice	High	Active in peer support (e.g., Alcoholics Anonymous)

OT—Therapeutic Unit.

**Table 3:** Jump performance parameters (median with lower; upper quartile) for Non-addicted and addicted individuals, including Z-values, *p*-values, and Effect sizes.

Measure	Non-Addicted	Addicted	Z-Value	*p*-Value	Effect Size
Jump Height [cm]	25.9 (19.5; 30)	22.7 (20.1; 26.55)	1	0.31	0.14
Power Peak [kW]	3.42 (3.09; 3.89)	3.14 (2.75; 3.94)	0.59	0.55	0.08
Velocity Peak [m/s]	2.36 (2.12; 2.63)	2.28 (2.08; 2.55)	0.80	0.42	0.11

**Table 4:** Median (lower; upper quartile) values of key Functional Movement Screen (FMS) parameters in Non-addicted and addicted groups, including Z-values, *p*-values, and Effect sizes, where * denote significant differences (*p* < 0.05).

Parameter	Non-Addicted	Addicted	Z-Value	*p*-Value	Effect Size
Deep Squat [cm]	3 (2; 3)	3 (2; 3)	0.27	0.78	0.04
Hurdle Step Right Leg	2 (2; 2)	2 (1; 2)	1.15	0.24	0.17
Hurdle Step Left Leg	2 (2; 2)	2 (2; 2)	0.69	0.48	0.1
Hurdle Step Overall Score	2 (2; 3)	2 (1; 2)	1.43	0.15	0.21
In-Line Lunge Right Leg	2 (2; 3)	2 (2; 2.5)	0.88	0.37	0.13
In-Line Lunge Left Leg	2.5 (2; 3)	2 (2; 2)	1.61	0.10	0.23
In-Line Lunge Overall Score	2 (2; 3)	2 (2; 2)	1.49	0.13	0.22
Shoulder Mobility Hand Length [cm]	19 (17; 20.5)	20 (19; 20)	0.83	0.40	0.12
Shoulder Mobility Right Hand Distance	2.5 (2; 3)	2 (1; 3)	1.26	0.20	0.18
Shoulder Mobility Left Hand Distance	3 (2; 3)	2 (1; 3)	2.36	0.01 *	0.34
Active Straight Right Leg Raise	3 (2; 3)	2 (2; 3)	1.74	0.08	0.25
Active Straight Left Leg Raise	3 (2; 3)	3 (2; 3)	1.13	0.25	0.16
Active Straight Leg Raise Score	3 (2; 3)	2 (2; 3)	1.60	0.10	0.23
Composite Raw Score	16.5 (15; 18)	15 (13; 16)	2.25	0.02 *	0.33

**Table 5:** Median (lower; upper quartile) handgrip strength scores across different arm positions in Non-addicted and addicted individuals, including Z-values, *p*-values, and Effect sizes.

Arm Position	Non-Addicted	Addicted	Z-Value	*p*-Value	Effect Size
90° Right Hand	49 (40; 52)	52 (48; 56)	−1.50	0.13	−0.22
90° Left Hand	49 (42; 52.5)	52 (46; 55)	−1.09	0.27	−0.16
Alongside Right Hand	51 (45; 57.25)	51 (48; 56)	0.14	0.29	0.02
Alongside Left Hand	50 (43; 56.25)	52 (46; 58)	−1.05	0.29	−0.15

**Table 6:** Median (lower; upper quartile) for reaction time and number of switched-off lights for Non-addicted and addicted groups, including Z-values, *p*-values, and Effect sizes.

Measure	Non-Addicted	Addicted	Z-Value	*p*-Value	Effect Size
Reaction Time [s]	0.48 (0.42; 0.53)	0.49 (0.47; 0.52)	0.37	0.70	0.05
Switched-off Lights [n]	72 (65.75; 78)	70 (68; 73.63)	0.30	0.76	0.11

**Table 7:** Median (lower; upper quartile) scores for psychological measures in Non-addicted and Addicted individuals, including Z-values, *p*-values, and Effect sizes.

Measure	Non-Addicted	Addicted	Z-Value	*p*-Value	Effect Size
Problem-Focused Coping	17 (14.75; 19)	17 (14.5; 18.5)	0.18	0.85	0.03
Emotion-Focused Coping	15 (12; 18)	16 (14.5; 18.5)	−0.62	0.52	−0.09
Avoidant Coping	6 (4; 7)	7 (6; 9.5)	−1.94	0.05	−0.28
Self-Critical Scale (SCS)	3.3 (3; 3.82)	3.3 (3; 3.6)	0.57	0.56	0.08
PHQ-9—Depression	3.5 (1.75; 10.25)	4 (1.5; 9.5)	−0.29	0.76	−0.04
PSS-10—Perceived Stress	20 (16.75; 23)	20 (18; 25)	−0.29	0.76	−0.04

**Table 8:** Key Spearman correlation (rho) values in addicted and non-addicted groups, showing only significant (*p* < 0.05) or trend-level (*p* < 0.10) associations with 95% confidence intervals (CIs). Confidence intervals were computed using the Fisher z-transformation, providing an estimate of the precision of each correlation. Narrower CIs indicate greater reliability, whereas wider intervals reflect higher uncertainty in the estimated relationship.

Physical Measure	Psychological Outcome	Addicted Group Rho (*p*-Value) 95% CI (Lower, Upper)	Non-Addicted Group Rho (*p*-Value) 95% CI (Lower, Upper)	Interpretation
Deep Squat [cm]	PSS-10	−0.46 (0.047) (−0.76, −0.01)	−0.18 (0.45) (−0.518, 0.207)	Better squat depth associated with lower stress (addicted)
Hurdle Step Left Leg	Emotion-Focused Coping	0.46 (0.048) (0.01, 0.76)	0.46 (0.048) (0.10, 0.71)	Better functional movement linked to adaptive coping
Alongside right handgrip strength [kg]	PHQ-9	0.47 (0.043) (0.02, 0.76)	−0.46 (0.049) (−0.71, −0.10)	Opposite associations in addicted vs. non-addicted groups
Peak Jump Velocity [m/s]	Avoidant Coping	−0.41 (0.078) (−0.73, 0.05)	−0.30 (0.21) (−0.60, 0.08)	Trend: stronger/faster jump linked to less avoidant coping
Reaction Time [s]	Avoidant Coping	0.45 (0.051) (−0.01, 0.75)	0.12 (0.74) (−0.26, 0.47)	Trend: slower reaction associated with more avoidant coping

## Data Availability

The datasets presented in our article are not readily available because the data are part of an ongoing study. Therefore, access is currently restricted. Requests to access the datasets can be directed to Michalina Błażkiewicz.
